# Mesenteric panniculitis of the sigmoid colon: a case report and review of the literature

**DOI:** 10.1186/1752-1947-1-108

**Published:** 2007-10-02

**Authors:** Angel I Popkharitov, Georgi N Chomov

**Affiliations:** 1 Department of Surgery, Thracian University, Medical Faculty, 11 Armeiska str, Stara Zagora 6000, Bulgaria; 2 Department of Pathology, District Hospital, Stara Zagora 6000, Bulgaria

## Abstract

**Introduction:**

Mesenteric panniculitis of the sigmoid colon is a rare occurrence in surgical practice. The aim of this article is to present a case of mesenteric panniculitis of the sigmoid colon and a short review of the literature.

**Case presentation:**

We reviewed the hospital record of a 63-year-old man who presented with a palpable mass in the left abdomen and clinical signs of a partial bowel obstruction. The pre-operative impression was a possible cancer of the sigmoid colon. A laparotomy was performed through a midline incision. The mesentery was found to be markedly thickened, constricted and puckered. The normal architecture of the adipose tissue had been lost and replaced with an irregular nodular mass. The microscopic pathologic sections demonstrated a chronic reactive inflammatory process with an exuberant proliferation of fibroblasts and fibrocytes. The adipose tissue contained scattered areas of steatonecrosis with foci of lipid-laden macrophages, lymphocytes and plasma cells. The sigmoid colon and its mesocolon were resected. The postoperative course was uneventful and the patient was discharged in good condition, and followed up for the next two years.

**Conclusion:**

Mesenteric panniculitis of sigmoid is an extremely rare entity of unknown origin in which the normal architecture of the mesentery is replaced by fibrosis, necrosis and calcification. On gross examination the alterations may be mistaken for a neoplastic process. A frozen section may be necessary for confirmation of the diagnosis. When the advanced inflammatory changes became irreversible and bowel obstruction occurs, resection may be indicated.

## Introduction

Mesenteric panniculitis (MP) is a benign fibro-inflammatory process involving adipose tissue of the mesentery characterized by the presence of fat necrosis, chronic inflammation and fibrosis. It is known by several terms including sclerosing mesenteritis and mesenteric lipodystrophy. Although the disease occurs rarely, during recent years there have been a number of reports which suggests a growing interest in this clinical entity [1-7]. We present a rare case of MP of the sigmoid colon and hope to encourage interest in further study and research.

## Case presentation

**Figure 1:**
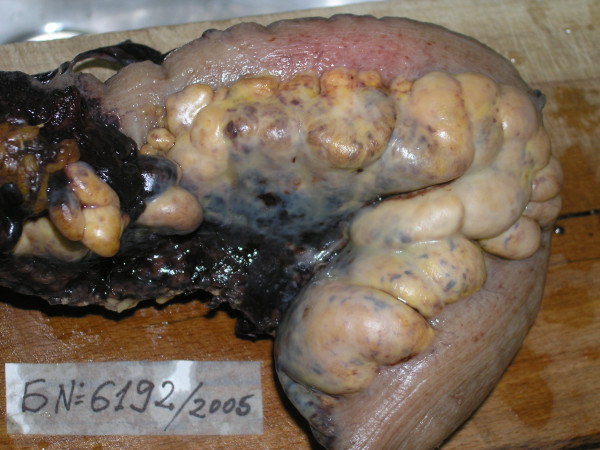
Photograph of the removed specimen shows the pattern of operative finding.

A 63-year-old man was admitted to our hospital with a two month history of general weakness, intermittent left lower quadrant abdominal pain, weight loss, constipation and signs of partial bowel obstruction. Physical examination revealed a palpable tender mass in the left abdomen. There was no history of melena or mucus. The overlying skin and subcutaneous tissue were unremarkable. A barium enema showed narrowing of the distal sigmoid colon. Colonoscopy revealed inflammatory changes of the sigmoid colon – the mucosa was erythematous and edematous, but there were no colonoscopic features of Crohn's colitis or ulcerative colitis. Computed tomography was negative except for a small amount of free fluid present in the abdomen. Laboratory findings were normal. The pre-operative impression was of a possible cancer of the sigmoid colon although the pre-operative investigations were not conclusive, particularly because no mucosal mass was identified. Laparotomy was performed through a midline incision. The mesentery of the sigmoid colon was markedly thickened, constricted and puckered. The color varied from yellow to red-brown suggestive of fat necrosis. The normal architecture of the adipose tissue was lost and was replaced with an irregular nodular mass involving appendices epiploicae of the colon. There was a moderate amount of serous effusion in the peritoneal cavity. No enlarged lymph nodes were identified. On macroscopic pathological evaluation the mesenteric mass was composed of areas of pale yellow and reddish-brown color with focal hemorrhage. The blood vessel walls were thickened and congested. The bowel wall adjacent to the mesenteric mass was thickened, edematous and markedly congested (figure 1).

**Figure 2:**
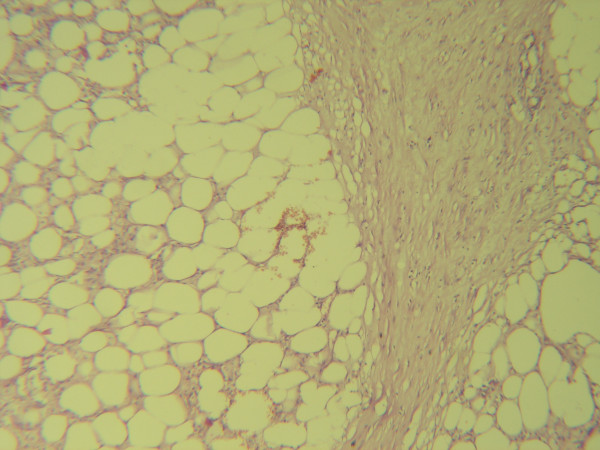
Histological section demonstrating zone of significant proliferation of fibroblasts, fibrocytes, collagenisation/H&E × 63/.

**Figure 3:**
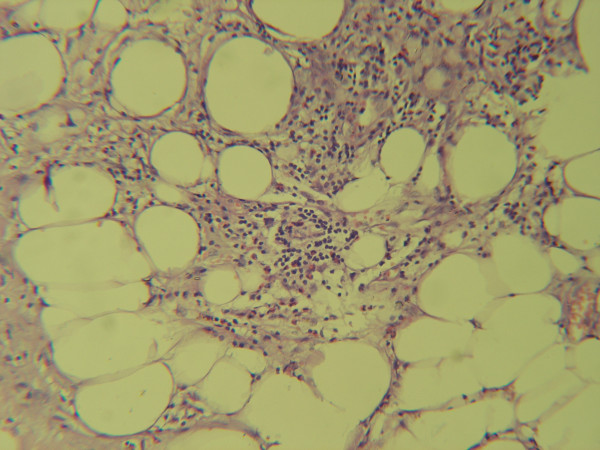
The fat tissue with scattered areas of necrosis, infiltrates of lipid-laden macrophages, lymphocytes and plasma cells/H&E × 250/.

Microscopic pathologic sections from the involved mesentery demonstrated a chronic reactive inflammatory process with exuberant proliferation of fibroblasts, fibrocytes and fibrosis (figure 2). Scattered areas of steatonecrosis and lipid-laden macrophages, lymphocytes and plasma cells (figure 3) were present. On microscopic examination of the sigmoid mucosa there were no inflammatory changes.

The postoperative course was uneventful, and the patient was discharged ten days after the operation in good condition. The patient's state of health was followed up for two years during which time he remained asymptomatic.

## Discussion

Mesenteric panniculitis is known in the literature by a variety of terms – "mesenteric manifestation of Weber-Christian disease" – introduced by Herrington and associates [8], "Isolated lipodystrophy"- a term used by Crane and al. [9], "mesenteric lipogranuloma"- by Weeks and co-workers [10], and "retractile mesenteritis"- by Jra [11]. Whether all these terms represent histologic variants of the same or related process of a single clinical entity is not entirely clear. The essential feature of the disease is the pathologic microscopic similarity between the affected fatty tissue and that seen in Weber-Christian disease.

MP affects predominantly the mesentery of the small intestine. This process rarely involves the large intestine. According to Wexner and Attiyeh [12] there have been 122 cases of MP described in the literature and only three involved the mesentery of the colon. These authors reported two cases of MP of the sigmoid colon. According to a literature review of Karentzos and co-workers [13] in 1990, only 5 of 124 cases of MP involved the mesentery of the sigmoid colon. Herington et al. [8] described two cases of MP; one involved the mesentery of the sigmoid colon. The expression "mesenteric manifestation of Weber-Christian disease" in our opinion most closely corresponds to the clinical and pathologic findings in the case we describe in this report. Emory and co-workers [14] reviewed 84 cases coded as mesenteric lipodystrophy, MP, retractile mesenteritis and sclerosing mesenteritis. In nineteen of them the mesocolon was involved. The authors suggest that the numerous of terms appear to represent histologic variants of one clinical entity. The most consistent histologic findings in that study were the presence of fibrosis and a chronic inflammatory infiltrate. Therefore they considered "sclerosing mesenteritis" as the most appropriate diagnostic term. Ogden and al. [15] reviewed 27 cases of MP which involved the mesentery of the small bowel. The patients presented with troublesome problems and were all diagnostically challenging. The most common preoperative diagnoses considered were pancreatic cyst, chronic pancreatitis, various types of intra-abdominal cancer and retroperitoneal tumor. The diagnosis was correct preoperatively in only two of the 27 cases. Ikoma and co-workers [16] reported one case of retractile mesenteritis of the rectosigmoid colon and reviewed 52 cases of the large bowel collected from the literature. The diagnosis was only able to be made at the time of laparotomy in 90.4% of the patients. The mass involving the colon was resected in 59.6% of cases. In our case we also had preoperative diagnostic difficulty. The clinical changes imitate a neoplasm of the sigmoid colon. The standard diagnostic methods which we performed were not helpful in establishing the diagnosis. Only gross macroscopic and microscopic examination of the surgically removed specimen clarified the diagnosis.

The etiology and pathogenesis of the disease are very obscure. Various factors such as blunt abdominal trauma or prior surgery, cold, different drugs, vasculitis, vitamin deficiency, autoimmune processes and allergic disease have been suggested as possible causes. Some of the cases of MP were associated with abdominal tuberculous lymphadenitis [17]. The association of autoimmune hemolytic anemia with MP suggests a possible relationship of an immune mechanism in the pathogenesis of the disease [18].

The diagnosis is frequently made by abdominal exploration. A frozen section may be necessary for confirmation of the diagnosis. There is no specific treatment for MP. There are reports of response to steroids [2], immunosuppressive therapy, antibiotics [18], tamoxifen [19]. Although some authors advise no radical surgical treatment [5,13,20], we believe this restriction should be limited only to cases of massive involvement when the removal of the affected parts is impossible and hazardous. In our opinion resection (when it is possible) is appropriate intraoperative practice in spite of the benign nature of the disease. This is especially in cases of bowel obstruction.

## Conclusion

MP of sigmoid is extremely rare clinical entity. On gross examination, the alterations may be mistaken for a neoplastic processes. A frozen section study may be warranted. The intraoperative findings are very typical and once seen the picture is not easily forgotten. There is no specific treatment. Medical treatment may consist of therapy with anti inflammatory or immunosuppressive agents. When the advanced inflammatory changes became irreversible, and especially in case of bowel obstruction, we recommend partial resection.

## Abbreviations

MP = Mesenteric panniculitis.

## Competing interests

The author(s) declare that they have no competing interests.

## Authors' contributions

AP participated in the sequence alignment, explored all possible sources for the references and drafted the definitive version of this manuscript.

GC had a substantial contribution of the review of the histological section of the case. He performed the photos and their interpretation.

All authors read and approved the final manuscript

## Consent

Written informed consent was obtained from the patient for publication of this study.
